# Endoluminal Vacuum Therapy (EVT) for the Treatment of Rectal Perforation Following Cleansing Enema Application

**DOI:** 10.7759/cureus.35939

**Published:** 2023-03-09

**Authors:** Alper Sozutek, Ekin Y Tas, Kemal Yener, Julia Ozcomert

**Affiliations:** 1 Gastroenterological Surgery, Health Sciences University, Adana City Training and Research Hospital, Adana, TUR; 2 Gastroenterological Surgery, Health Sciences University, Adana City Training and Research Hospital, Adana , TUR

**Keywords:** colon and rectal surgery, endoscopic management, fleet enema, rectum perforation, endoluminal vacuum

## Abstract

Traumatic rectal injuries (TRIs) are challenging for surgeons because of their high morbidity and mortality. Considering the well-known predisposing factors, enema-associated rectal perforation seems to be the most overlooked entity that leads to devastating rectal injuries. A 61-year-old man with a three-day history of painful swelling around his perirectal area after enema application was referred to the outpatient clinic. CT demonstrated the presence of a left posterolateral rectal abscess compatible with an extraperitoneal rectal injury. Sigmoidoscopy revealed the perforation started 2 cm above the dentate line with a diameter of 10 cm and a depth of 3 cm. Endoluminal vacuum therapy (EVT) and laparoscopic sigmoid loop colostomy was performed. The patient was discharged after removing the system on postoperative Day 10. On his follow-up, the perforation side was totally closed and pelvic abscess was completely resolved two weeks after his discharge. EVT appears to be a simple, safe, well-tolerated and cost-effective therapeutic procedure in the management of delayed extraperitoneal rectal perforations (ERPs) with large defects. To our knowledge, this is the first case that reveals the potency of EVT in the management of a delayed rectal perforation associated with an uncommon entity.

## Introduction

Traumatic rectal injuries (TRI) remain a challenging entity for the surgeons because of their unpredictable extent of the damage through the rectal tract and around the perirectal area. Many risk factors leading to TRI have been reported in the current literature. Among these, enema application is an uncommon entity [1,2]. The exact incidence of enema-associated rectal perforation is unknown and likely to have been underreported. In spite of its rarity, it is relatively associated with higher morbidity and mortality than other risk factors because it has a potential to cause devastating intra/extraperitoneal rectal perforations (ERPs) resulting in large defects with delayed intra-abdominal/pelvic sepsis. Comparing with intraperitoneal rectal perforations, the management of ERP is still a subject of debate [3]. Evolving endoscopic procedures like stenting or clipping of the perforation have recently been accepted as the first-line treatment of ERP [3,4]. However, the availability and expertise in this field limit their widespread usage. In addition, endoscopic interventions usually may fail either closing large defects or controlling the source of abscess, particularly in a delayed condition. 

Endoluminal vacuum therapy (EVT) is a modification of conventional vacuum-assisted wound closure (VAC) system that has been adapted for internal use, which yields promising results in the management of anastomotic leakage after rectal surgery [5]. In spite of the fact that EVT is currently not a standard therapy for ERP, successful outcomes of few cases with ERP have recently been reported [6]. However, EVT has been immediately used after diagnosis in these cases, so the clinical benefit of EVT in a delayed diagnosis with pelvic abscess remained unclear. Considering the paucity of data regarding the feasibility of EVT in the management of such a circumstance, contributing every data is essential to further elucidate its efficacy in the management of ERP. To our knowledge, the present case is the first one in the current literature that reveals the effectiveness of EVT in the management of a delayed rectal perforation.

## Case presentation

**Figure 1:**
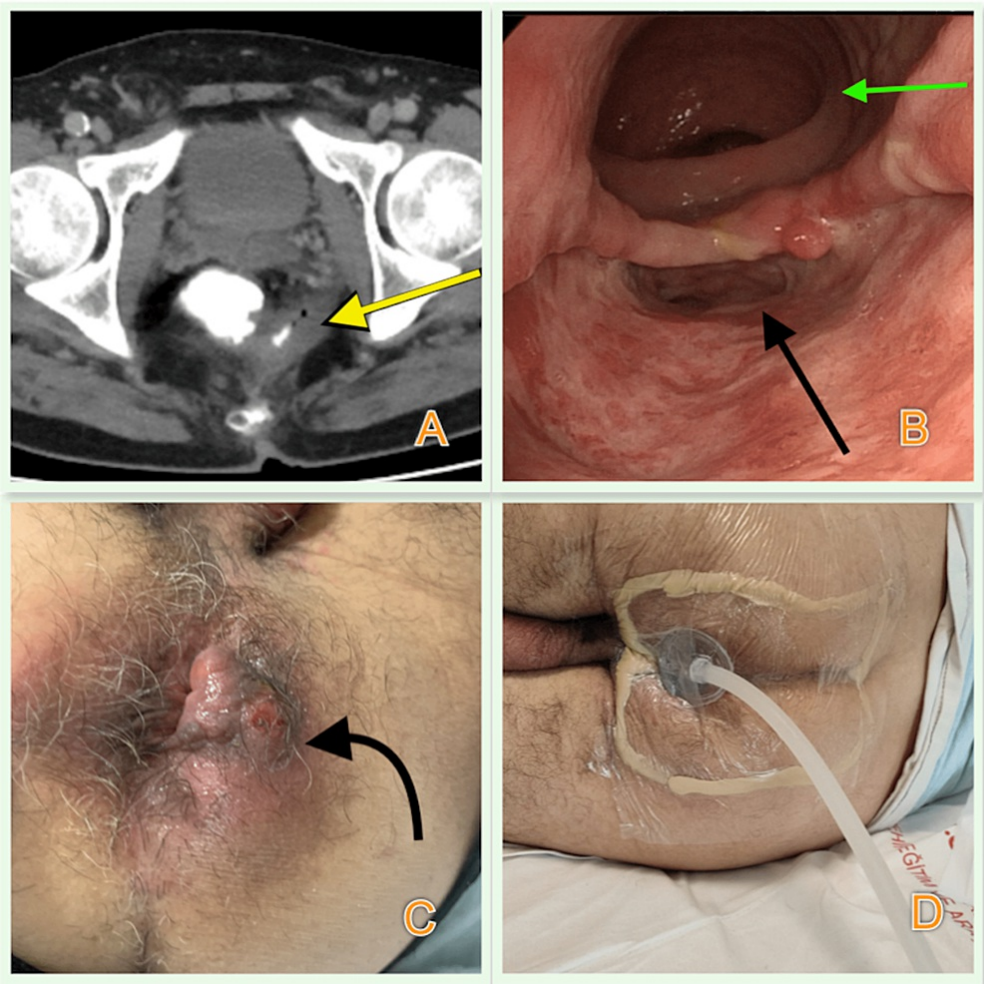
View of initial imaging studies, clinical presentation of the patient, and the demonstration of EVT A) CT view of ERP involving the posterolateral wall of the rectum (marked with yellow arrow); B) The view of rectal perforation on colonoscopy (the perforation area is marked with black and normal lumen is marked with green arrow); C) The view of perianal abscess in lithotomy position; D) EVT application with open-pore polyurethane sponge EVT: Endoluminal vacuum therapy; ERP: Extraperitoneal rectal perforation

**Figure 2:**
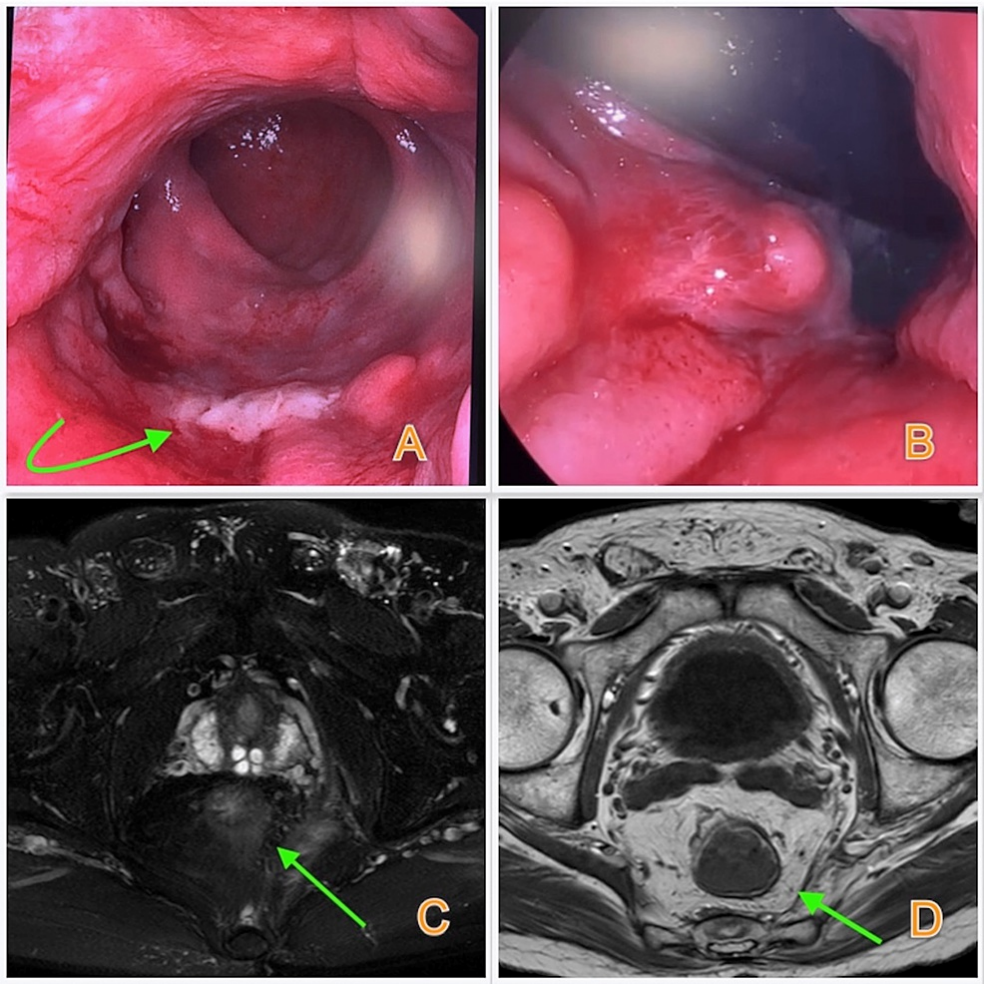
View of colonoscopy and MRI after EVT A) Control colonoscopy revealing the perforation side totally closed with a granulation tissue (marked with green arrow); B) Retrovert view of the granulation tissue; C-D) MRI that confirms total resolution of the abscess (marked with green arrows) MRI: Magnetic resonance imaging; EVT: Endoluminal vacuum therapy

A 61-year-old man with a three-day history of painful swelling around his perirectal area after enema application was referred to our clinic. He expressed the anal pain was precipitated by the first application of enema. He was preparing for colonoscopy for routine colorectal cancer screening program. His medical history was unremarkable. At admission, his vital signs were normal except a slight fever (37.6°C). On physical examination, painful fluctuating abscess located at the left lateral wall of the anus was observed. On digital examination, more than a half of the posterior wall of the rectum had perforated. His abdominal examination was normal. Laboratory parameters revealed mild leukocytosis (14,200/mm^3^) and increased C-reactive protein (72 mg/dL). Abdominal CT demonstrated the presence of a left posterolateral rectal abscess extending to the mesorectum compatible with an extraperitoneal rectal injury. On sigmoidoscopy, the perforation started from 2 cm above the dentate line extending up to 10 cm along the rectum with a diameter of 10 cm and a depth of 3 cm (Figure 1). The pouch was irrigated with saline. Because of the size, fragility and tightness of the injured tissue, over-scope clip was determined to fail to close the defect. Hence, the surgery was planned. On surgery, perianal abscess drainage and laparoscopic sigmoid loop colostomy was performed. Subsequently, a 10 cm-long modified VAC sponge was placed into the cavity via colonoscopy and the system was continuously set at -40 mmHg. After the procedure, the patient completed five days course of oral ciprofloxacin 500 mg every 12 hours and metronidazole 500 mg every eight hours. The inflammatory parameters decreased after postoperative Day 2. The drainage system was renewed every 72 hours for three times. The system was well-tolerated by the patient. The postoperative course was uneventful. The patient was discharged after removing the system on postoperative Day 10. The rectum was checked by both sigmoidoscopy and pelvic magnetic resonance (MR) two weeks after his discharge. The perforation side was totally closed and pelvic abscess was completely resolved (Figure 2). Upon these findings, his colostomy was closed after one week. After six months of follow-up, the patient remains free from complications and sequelae. 

 

## Discussion

Enemas are injections of fluid used to clean distal bowel before colonoscopy as well as to treat constipation or inflammatory bowel diseases. In spite of the fact that enema application seems to be a relatively innocent and easy procedure, clinicians should be aware that it is a penetrating trauma to the rectum. Considering that most of the patients perform this application on their own, careless practices may lead to life-threatening complications such as rectal perforation, as in the present case. The rarity of enema-related complications in the current literature should not mislead the clinician since this subject might be underestimated because of underreporting [1]. Hence, another aim of this paper is to increase awareness about the correct use of enema. Rectal perforation is frequently associated with the sudden pressurized delivery of the enema, particularly when obstructive mass, inflammation, or anastomotic stenosis of the rectum exists. In addition, rough insertion of the device tip may injure the rectum because of the anorectal angle and localized weakness of the rectal wall, as in our case, so the patient should be warned about the position during enema application. Keeping the enema tip in the center of bowel lumen after insertion is essential to avoid any injury to the rectal wall [2]. If an injury occurs at the extraperitoneal part of the rectum, the patient may not feel pain immediately because the mucosa above the dentate line is insensitive and there is a lack of intraperitoneal fecal contamination as well. The pain starts particularly at the posterior side of the anus when the injected liquid becomes irritating into the perirectal space. Moreover, a perianal abscess or pelvic sepsis may develop days later following application, as in the present case. Therefore, the patient should be warned to consult a clinician when unexpected anal pain or bleeding occurs after enema application to avoid delay in diagnosis and the initiation of proper treatment strategies. 

The lower one-third of the rectum is located extra-peritoneally, so there is a low possibility of fecal contamination into the abdominal cavity which is a condition that does not require prompt surgery. However, comparing with intraperitoneal injuries, the optimal treatment method is more controversial. [3] Conservative management, including bowel rest and systemic antibiotherapy, may be amenable for hemodynamically stable patients with minimal rectal perforation that is limited to a small retroperitoneal area. However, when the width of the defect is greater than 2 cm, fecal contamination leads to pararectal abscess and pelvic sepsis. Recently, contemporary endoscopic techniques including stents, fibrin glue, double-pig tail catheter placement or through/over-the-scope clips have been proposed to be the first-line therapeutic approach for these conditions instead of surgery [4]. However, the availability and expertise in this field limit their widespread usage. Thus, surgery is still the first approach in many centers. Based on the available evidence, primary repair concomitant with fecal diversion is the most performed surgical intervention. Unfortunately, all these approaches may fail to close large defects with poor tissue quality and when a severe inflammation is present as well. Moreover, because of the unknown width or depth of the defect, these approaches may fail to drain the cavity that may be resulting in persistent perianal abscess. Therefore, a therapeutic approach that ensures closing the perforation area while reducing the source of sepsis in a controlled manner can be the most appropriate treatment approach. Since EVT offers these advantages, we decided to perform it in the present case. Laparoscopic sigmoid loop colostomy was also performed due to avoid fecal contamination that leads to block the drainage system and the treatment period was uncertain, as well. 

EVT is a modification of conventional VAC system that has been adapted for internal use. This system provides continuous or intermittent drainage via an open-pored polyurethane sponge that decreases bacterial contamination, secretion, and local edema, thereby enhancing recovery by promoting perfusion and granulation of the injured tissue [4,6]. VAC therapy is effective at -125 mmHg; however, such a high negative pressure causes tissue necrosis around sealed mucosa [7]. For this reason, it is not licensed for rectal use, so commercially available endoluminal sponges have been introduced. However, the sponge can be easily prepared from the VAC sponge to reduce the cost. We achieved successful outcome by decreasing the negative pressure to -40 mmHg and well-tolerated by the patient. In our experience, EVT ensures a significant enhanced recovery of the perforation area in 15 days by promoting tissue granulation with a proper control the source of abscess. Another advantage of EVT that should be considered that it may provide shorten hospitalization when compared with other techniques. 

Sponge dislocation, persistent anal pain, and tissue necrosis due to the system being set at high negative pressure, discomfort due to the repeated colonoscopy and the placed system are the most frequent adverse events that can be observed during EVT [4,5]. Moreover, minor bleeding may occur while exchanging the sponge due to ingrowth of granulation tissue into the sponge. This condition can be easily controlled with conservative treatments [4]. The risk of fecal incontinence and rectal stenosis are still major concerns that needs to be clarified in the long term. None of these adverse events were observed either in our early or the late-term follow-up.

## Conclusions

We presented a case with delayed rectal perforation who was successfully treated by EVT. Despite the large defect, EVT ensured a significant short recovery period with proper control of the source of abscess. We are aware that this is an anecdotal case presentation, so it is difficult to make a definitive comment on the exact potential of EVT. However, our clinical outcome encourages us to suggest that EVT may be considered as a simple, safe, well-tolerated, and cost-effective therapeutic alternative procedure when endoscopic or surgical interventions fail. This suggestion should be investigated with further cases regarding this situation. 
